# Metabolic features of treatment-refractory major depressive disorder with suicidal ideation

**DOI:** 10.1038/s41398-023-02696-9

**Published:** 2023-12-15

**Authors:** Lisa A. Pan, Jane C. Naviaux, Lin Wang, Kefeng Li, Jonathan M. Monk, Sai Sachin Lingampelly, Anna Maria Segreti, Kaitlyn Bloom, Jerry Vockley, Mark A. Tarnopolsky, David N. Finegold, David G. Peters, Robert K. Naviaux

**Affiliations:** 1 Department of Psychiatry, University of Pittsburgh School of Medicine, Pittsburgh, PA USA; 2 School of Public Health, Department of Human Genetics, University of Pittsburgh School of Medicine, Pittsburgh, PA USA; 3 Panomics Mental Health Initiative, Pittsburgh, PA USA; 4 New Hope Molecular, LLC, Pittsburgh, PA USA; 5 The Mitochondrial and Metabolic Disease Center, University of California, San Diego School of Medicine, San Diego, CA USA; 6 Department of Neurosciences, University of California, San Diego School of Medicine, San Diego, CA USA; 7 Department of Medicine, University of California, San Diego School of Medicine, San Diego, CA USA; 8 Department of Pediatrics, University of Pittsburgh School of Medicine, Pittsburgh, PA USA; 9 Department of Pediatrics, McMaster University, Hamilton, Ontario Canada; 10 Department of Obstetrics, Gynecology & Reproductive Sciences, University of Pittsburgh, Pittsburgh, PA USA; 11 Department of Pediatrics, University of California, San Diego School of Medicine, San Diego, CA USA; 12 Department of Pathology, University of California, San Diego School of Medicine, San Diego, CA USA; 13 Present Address: New Hope Molecular, LLC, 750 Washington Rd, Suite 19, Pittsburgh, PA 15228 USA

**Keywords:** Depression, Pathogenesis

## Abstract

Peripheral blood metabolomics was used to gain chemical insight into the biology of treatment-refractory Major Depressive Disorder with suicidal ideation, and to identify individualized differences for personalized care. The study cohort consisted of 99 patients with treatment-refractory major depressive disorder and suicidal ideation (trMDD-SI *n* = 52 females and 47 males) and 94 age- and sex-matched healthy controls (*n* = 48 females and 46 males). The median age was 29 years (IQR 22–42). Targeted, broad-spectrum metabolomics measured 448 metabolites. Fibroblast growth factor 21 (FGF21) and growth differentiation factor 15 (GDF15) were measured as biomarkers of mitochondrial dysfunction. The diagnostic accuracy of plasma metabolomics was over 90% (95%CI: 0.80–1.0) by area under the receiver operator characteristic (AUROC) curve analysis. Over 55% of the metabolic impact in males and 75% in females came from abnormalities in lipids. Modified purines and pyrimidines from tRNA, rRNA, and mRNA turnover were increased in the trMDD-SI group. FGF21 was increased in both males and females. Increased lactate, glutamate, and saccharopine, and decreased cystine provided evidence of reductive stress. Seventy-five percent of the metabolomic abnormalities found were individualized. Personalized deficiencies in CoQ10, flavin adenine dinucleotide (FAD), citrulline, lutein, carnitine, or folate were found. Pathways regulated by mitochondrial function dominated the metabolic signature. Peripheral blood metabolomics identified mitochondrial dysfunction and reductive stress as common denominators in suicidal ideation associated with treatment-refractory major depressive disorder. Individualized metabolic differences were found that may help with personalized management.

## Introduction

Midlife mortality has been increasing in the US since 2000 [1]. This trend has many contributing causes, including increased suicide and drug overdose rates that have been referred to collectively as “deaths of despair” [2]. Suicides among teens and young adults are also rising in the US [3]. The aggregate effect in the US since 2014 has been the first decrease in the average life expectancy since 1959 [4]. This trend occurred even before the excess mortality caused by the COVID pandemic. Although many countries around the world have experienced similar upward trends in suicidality, other countries in Europe and South America have shown decreasing trends [5]. These national and regional differences underscore the importance of potentially modifiable psychosocial and socioeconomic factors in determining suicide risks. In this paper, we apply the new tools of metabolomics to characterize the chemistry associated with one component of this alarming trend in US mortality—treatment-refractory major depressive disorder with suicidal ideation.

Major depressive disorder (MDD) affects 16.1 million adults in the United States and costs $210 billion annually [6]. The worldwide point prevalence is 6% [7]. The risk of recurrence of depression after the first episode of MDD is 3–6 times the background population risk [8]. Most patients will have a recurring-remitting course with a median of 5 episodes over their lifetime. The risk of recurrence and the risk of suicide combine to create a growing social and medical challenge in nations around the world [9, 10]. Suicidal thoughts are experienced by the majority of patients with treatment-refractory depression [11] and 30% will attempt suicide at least once in their lifetime [12]. The rising tide of treatment-refractory depression and suicide has been seen as a growing epidemic in the US [13]. While single genes can play a role in certain rare forms of treatment-refractory depression [14], intensive search over the past 15 years has failed to identify any single gene that accounts for more than 1% of cases [15]. In addition, while human DNA has not changed in the past 20 years, the prevalence of depression has been increasing 10% per decade [16], and suicide mortality has increased by 33% since 1999 [17]. The toll from suicide has been increasing even faster among military veterans and is exacerbated by co-occurring post-traumatic stress disorder (PTSD) with or without depression [18]. These facts have underscored the importance of environmental factors leading to increased cellular and mitochondrial stress responses [19–21] and changing gene-environment interactions, including social and economic factors, in the pathophysiology of depression and suicide [22, 23]. New tools for assessing the biological impact of environmental stress are needed to help identify patients at greatest risk and provide new direction for research.

Metabolism and behavior are inextricably intertwined [24–26]. Metabolism represents the real-time interaction of genes and the environment. The emerging recognition that the brain controls metabolism through neuroendocrine, autonomic, immune, and microbiome circuits [27] provides a scientific rationale for the use of peripheral blood metabolomics for the discovery of novel biomarkers and pathophysiologic insights that may be diagnostic of functional changes in the brain-body system. Previous studies have shown that plasma metabolomics can be used as a diagnostic tool in studies of myalgic encephalomyelitis/chronic fatigue syndrome [28], schizophrenia [29], Gulf War Illness [30], response to treatment in autism spectrum disorder [24], and treatment-refractory MDD with suicidal ideation [14]. Metabolomics has also been shown to be useful prognostically in identifying patients at greatest risk for a recurrence of depression [25]. Emerging studies have shown that metabolism is regulated by factors that go beyond the concepts of substrate-product relationships, rate-limiting steps, and allosteric regulation. Whole pathways appear to be coordinately controlled, even when the products of those pathways have no obvious biochemical connection.

### Aims of the study

The aims of this study were: 1) to characterize the plasma metabolome of patients with severe depression and suicidal ideation, 2) to identify sex differences in the metabolic response to depression, and 3) to identify personalized differences in metabolism that might be used to tailor clinical management.

## Participants and methods

### Study design and IRB approvals

This study was approved by the University of Pittsburgh Investigational Review Board (IRB) under Dr. Pan’s projects PRO11120375, PRO14060600, and PRO12060048, and by the University of California, San Diego IRB under Dr. Naviaux’s project #140072. Signed informed consent was obtained from all participants. Samples were collected from 2014-2018 and stored at -80˚C until analysis. Fifty-eight plasma samples from healthy controls (30 males and 28 females) were collected at UCSD. Patient samples were matched by sex, age, and frozen storage time with healthy control samples. A total of 192 participants (*n* = 47 males and 53 females with trMDD-SI and 46 male and 47 female controls) were enrolled. Power analysis was used to estimate the minimum sample size. Based on effect size measured as a mean z-score difference of ≥ 0.6, α ≤ 0.05, and β ≤ 0.2 (power > 0.8), the minimum sample size was calculated to be 45 cases and 45 controls per single-sex group (https://sample-size.net/sample-size-means/).

### Patients and controls

Participants aged 18 to 70 years with depression unresponsive to known treatments (at least three maximum dose medication trials for at least 6 weeks each) were recruited by advertisement through the Clinical and Translational Science Institute Registry at the University of Pittsburgh or by clinical referral. Participants were compared with young adult healthy controls with no personal or first-degree relative history of psychiatric disorder or suicidal behavior. Participants were assessed with a structured psychiatric interview, including the Family Interview for Genetics Studies [31] at the time of referral to characterize depression course, comorbidity, family history, and history of trauma, psychosis, and anxiety. Participants completed the Antidepressant Treatment History Questionnaire [32], the Beck Depression Inventory (BDI) [33] and the Suicide Ideation Questionnaire [34]. Patients remained on current medications and remained in current treatment during the course of the study.

### Study procedures

Assessment consisted of a psychiatric interview, review of records, and administered self-reports at intake (characterized depression course (Beck Depression Inventory, BDI), suicidal ideation and behavior (Suicidal Ideation Questionnaire, Columbia Suicide History Form [35] and Beck Suicide Ideation Scale [36], comorbidity (anxiety, psychosis, substance use, attention disorders, DSM 5 Cross-Cutting Symptom Inventory) [37], and family history (Family Interview for Genetic Studies and 3 generation pedigree) [31]. A neurologic examination was completed by Dr. Pan. Blood and urine samples were analyzed by the Clinical Biochemical Genetics and Clinical Chemistry Laboratories of UPMC and Medical Neurogenetics, Inc., Atlanta, GA. If a specific inborn error of metabolism was suspected on the basis of the initial testing, the patient was referred to a biochemical geneticist for additional confirmatory testing. Upon receipt of results of testing, a follow-up appointment was scheduled for every affected participant to review results and provide additional referrals if needed. Participants with treatment-refractory depression returned for a second appointment to review results. At this appointment, BDI, Suicidal Ideation Questionnaire, and DSM 5 Symptom Inventory were repeated.

### Inclusion and exclusion criteria

Inclusion criteria for trMDD-SI cases were: ages 18 to 70 years with depression unresponsive to at least three maximum dose medication trials for at least 6 weeks each. Exclusion criteria for cases were: risk from lumbar puncture (e.g., coagulation disorder, brain mass, or traumatic brain injury), schizophrenia, drug or alcohol abuse in the 6 months prior to assessment. Inclusion criteria for healthy controls were: age- and gender-matched, healthy 18 to 70 years of age. Exclusion criteria for healthy controls were: personal diagnosis or a first-degree relative diagnosis of any mental health disorder, suicidal behavior, mental health treatment, or counseling.

### Metabolomics

Targeted, broad-spectrum, metabolomic analysis of 672 metabolites extracted from lithium-heparin anticoagulated plasma was performed by high-performance liquid chromatography, electrospray ionization, and tandem mass spectrometry (LC-MS/MS) as previously described [38] with minor modifications. Blood samples were collected at least 3 hours after a meal, between the hours of 8 am and 5 pm. A total of 448 of the 672 targeted metabolites were measurable in the plasma of both males and females. 100% of samples provided AUC data on these chemicals. There were no missing values, and no data were imputed. This targeted metabolomics platform interrogated 55 biochemical pathways and permitted analysis of many of the metabolites known to be core features of the cell danger response (CDR), which includes the integrated stress response (ISR) [21, 39, 40]. See Supplementary Information for additional details.

### FGF21 and GDF15

Human fibroblast growth factor 21 (FGF21) in plasma was measured using commercially available ELISA kit (BioVendor, cat# RD19108200R, Brno, Czech Republic) according to the manufacturer’s protocol. 60 μL of plasma from each patient was diluted 1:4 and 100 µL of the diluted sample was added to each well in duplicate. Standard curve range was from 30 pg/mL to 1920 pg/mL. Human growth differentiation factor 15 (GDF15) in plasma was measured using a commercially available ELISA kit (R&D systems, #SGD150) according to the manufacturer’s protocol. 50 μL of plasma from each patient was diluted 1:4 and 50 µL of diluted sample was added to each well in duplicate. High and low concentration controls (R&D systems, #QC21, Minneapolis, MN, USA) were added in each plate. Standard curve range was from 23.4 pg/mL to 1,500 pg/mL. Absorbance was measured at 450 nm using a POLARstar Omega plate reader (BMG Labtech, Cary, NC, USA). Missing values in a maximum of 15% of the samples were imputed using probabilistic principal component analysis (PPCA) [41].

### Statistical analysis

Demographic data were analyzed by t-tests or non-parametric Mann-Whitney U tests. Categorical data and 2 ×2 tables were analyzed by Fisher’s exact test. Area under the curve (AUC) data from metabolomics were log_2_ transformed, scaled by control standard deviations, and the resulting z-scores were analyzed by variable importance in projection (VIP) scores calculated by multivariate partial least squares discriminant analysis (PLSDA) in MetaboAnalyst [42, 43]. Mean decrease in accuracy (MDA) scores were calculated by random forest analysis from 5000 trees in R [44]. False discovery rates (FDRs) were calculated by the method of Benjamini and Hochberg [45] and Bayesian false discovery rates by Storey q value [46]. Significant metabolites had Mann-Whitney p values < 0.05, VIP > 0.9, and MDA > 0. These were grouped into biochemical pathways by the sum of their VIP or MDA scores to determine the rank-ordered significance of each pathway. Bubble impact plots were visualized in python. The percent impact was quantified on the x-axis as the sum of the MDA scores for metabolites in each pathway divided by the total MDA of metabolites from all pathways that reached the composite significance threshold of Mann-Whitney *p* values < 0.05, VIP > 0.9, and MDA > 0. Significance was quantified as the hypergeometric *p* value and plotted on the y-axis. Personalized metabolome differences were defined as metabolites that had Z-scores >+2.0 or <-2.0 compared to the control range and multivariate VIP scores < 1.5. Metabolites that were abnormal and contributed to the diagnosis of trMDD-SI had absolute Z-scores greater than 2.0 and VIP scores > 1.5. See Supplementary Information for additional methods.

## Results

### Participant characteristics

**Fig. 1:**
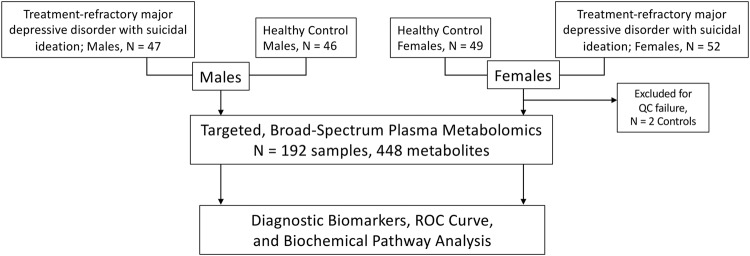
Study design. Plasma metabolomic analysis was performed in a cohort of *N* = 47 males with treatment-refractory major depressive disorder and suicidal ideation (trMDD-SI), and 46 healthy control males, and *N* = 52 females with trMDD-SI and 47 healthy control females.

Samples from a total of 99 males and females with a history of treatment-refractory major depressive disorder and suicidal ideation (trMDD-SI) and 93 healthy controls were analyzed (Fig. 1, Table 1). The median patient age was 29 years (IQR 22–42). Most patients had a history of major depressive disorder dating from their teens with a mean age of onset of 13 ± 6 years. Additional psychiatric diagnoses were common. Generalized anxiety disorder (GAD) occurred in 70–75%. Bipolar disorder (BP) occurred in 30–35%. Female patients in this cohort had an increased rate of post-traumatic stress disorder (PTSD) of 40% compared to 21% in males (Table 1, *p* = 0.05). Males had a higher rate of obsessive-compulsive disorder (OCD) of 21% compared to 6% in females (Table 1, *p* = 0.03). Nearly half had a history of one or more past suicide attempts and approximately 65% of patients had received inpatient psychiatric care (Table 1). Tobacco use was higher in males with suicidal ideation than in male healthy controls. There was a trend toward increased tobacco use in females, but this was not significant. No significant differences in body mass index (BMI) were present. Prescribed medications were highly personalized. Over 130 medications were used by the 192 subjects in this study (Supplementary Table S9). This diversity of medications ensured that the metabolomic differences observed were driven by the biology that was shared by patients with suicidal ideation, and not by any single medicine or class of medicines. All patients received psychotherapy tailored to their individual needs (Table 1).

**Table 1:** Participant characteristics.

	MALES			FEMALES			trMDD-SI
	trMDD-SI Mean ± SD (Range)	CONTROLS Mean ± SD (Range)	*p*	trMDD-SI Mean ± SD (Range)	CONTROLS Mean ± SD (Range)	*p*	Males vs. Females p
**Subjects enrolled**	47	46		52	47		
**Age in years** **(median)**	29 (IQR 23–41)	27 (IQR 22–44)	0.96	29 (IQR 24–36)	29 (IQR 22–42)	0.37	0.48
**Plasma samples analyzed**	47 (100%)	46 (100%)	0.99	52 (100%)	47 (96%)	0.99	0.99
**Body Mass Index** **(BMI)**	27 ± 5.2 (19–40)	27 ± 4.2 (20–38)	0.41	27 ± 7.3 (19–56)	24 ± 2.9 (20–34)	0.11	0.99
**Tobacco use**	20/47 (43%)	7/40 (17%)	0.02*	16/52 (31%)	8/48 (17%)	0.11	0.29
**BDI score**	29 ± 9.4 (12–50)	2.1 ± 2.4 (0–7)^a^	0.0001*	30 ± 9.6 (5–53)	0.7 ± 1.2 (0–5)^b^	0.0001	0.99
**SIQ score**	33 ± 20 (4–90)	3.1 ± 3 (0–9)^a^	0.0001*	37 ± 18 (4–71)	1.2 ± 1.5 (0–5)^b^	0.0001	0.17
**Age of 1**^**st**^ **episode of MDD**	13 ± 5.6 (1–25)	n/a		13 ± 6.1 (2–34)	n/a		
**Duration of MDD** **(years)**	20 ± 14 (1–63)	0	0.0001*	18 ± 8.4 (7–46)	0	0.0001*	0.39
**Lifetime episodes of depression** **(median)**	1 (IQR 1-2)	0	0.0001*	1 (IQR 1-5)	0	0.0001*	0.99
**Longest episode of MDD** **(years)**	16 ± 13 (0.2–63)	0	0.0001*	13 ± 9.4 (0.1-40)	0	0.0001*	0.19
**Additional diagnoses**							
**GAD**	33 (70%)	0	0.0001*	39 (75%)	0	0.0001*	0.65
**PTSD**	10 (21%)	0	0.001*	21 (40%)	0	0.0001*	0.05*
**BP**	14 (30%)	0	0.0001*	18 (35%)	0	0.0001*	0.67
**OCD**	10 (21%)	0	0.001*	3 (6%)	0	0.24	0.03*
**Number or prescription medications**	3.0 ± 2.6 (0–11)	0.6 ± 1.1 (0–5)	0.0001*	3.5 ± 2.2 (0–9)	0.2 ± 0.6 (0–3)	0.0001*	0.13
**Psychotherapy**^**c**^	47 (100%)	0	0.0001*	52 (100%)	0	0.0001*	0.99
**History of inpatient admissions**^**d**^	30 (64%)	0	0.0001*	33 (63%)	0	0.0001*	0.99
**History of past ECT**	14 (30%)	0	0.0001*	11 (21%)	0	0.0006*	0.36
**History of attempted suicide**	19 (40%)	0	0.0001*	24 (47%)	0	0.0001*	0.44
**Ethnicity**							
**Caucasian**	43 (91%)	38 (83%)	0.23	44 (85%)	42 (86%)	0.99	0.36
**African American**	1 (2%)	1 (2%)		2 (4%)	2 (4%)		
**Asian**	2 (4%)	5 (11%)		3 (6%)	3 (6%)		
**Mixed ethnicity**	1 (2%)	2 (4%)		3 (6%)	2 (4%)		

*TR-MDD-SI* treatment-refractory major depressive disorder with suicidal ideation, *BDI* Beck depression inventory, *SIQ* suicide ideation questionnaire, *ECT* electroconvulsive therapy, *GAD* generalized anxiety disorder, *PTSD* post-traumatic stress disorder, *BP* bipolar disorder, *OCD* obsessive compulsive disorder, *IQR* interquartile range.

*Significant *p* value ≤ 0.05. ^a^Based on 16 healthy male controls. ^b^Based on 20 healthy female controls. ^c^Included cognitive behavioral therapy (CBT), dialectical behavioral therapy (DBT), interpersonal psychotherapy (IPT), eye movement desensitization and reprocessing (EMDR), pastoral counseling, and/or other forms of psychotherapy tailored to patient needs. ^d^Inpatient neuropsychiatric admissions.

### Plasma metabolomics overview

**Fig. 2:**
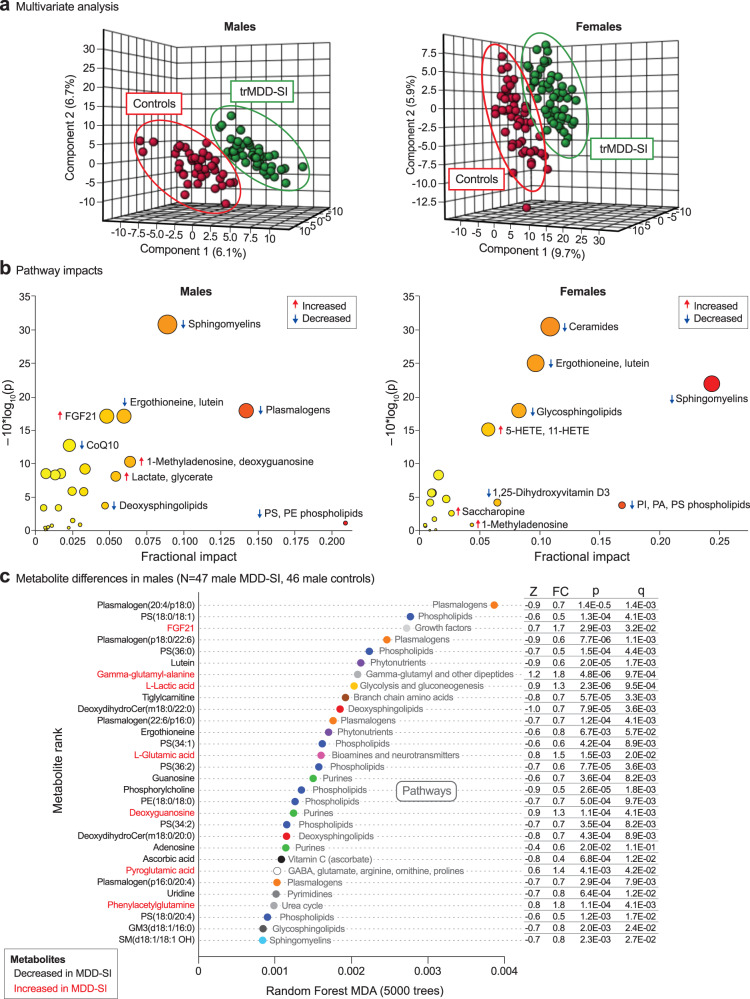

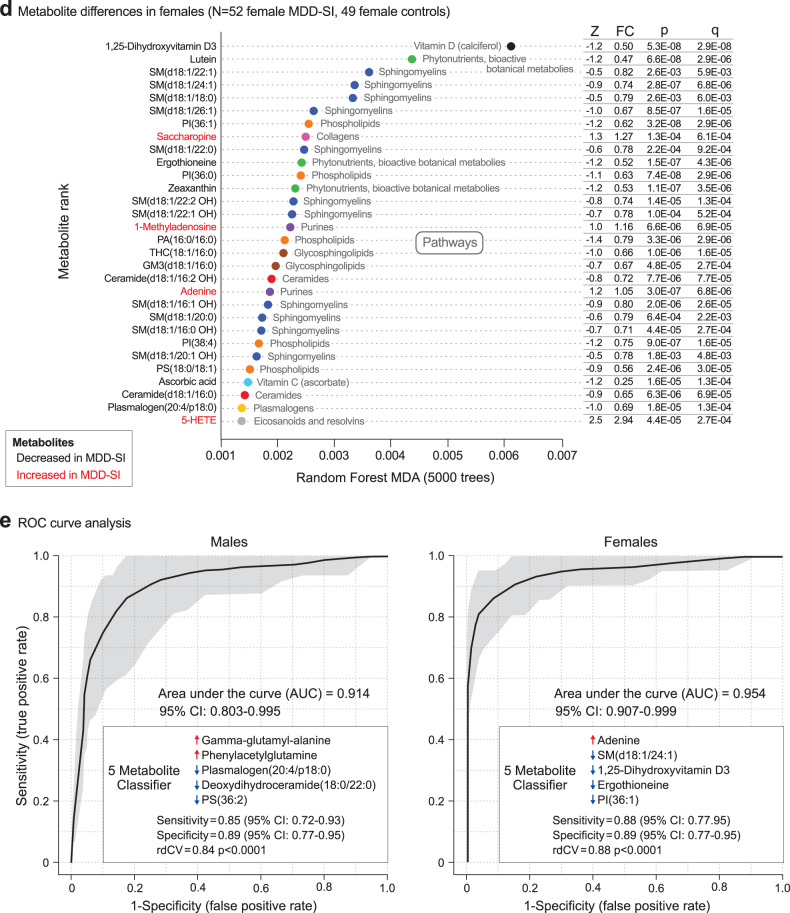
Metabolomic analysis of treatment-refractory major depressive disorder with suicidal ideation. **a** Multivariate analysis, males and females. 3-dimensional projection of partial least squares discriminant analysis. **b** Metabolic pathway impact bubble plots. The size of the bubble is proportional to the *p* value significance. The color from yellow to red is proportional to the percent impact calculated by random forest analysis, **c** Metabolite differences in males, **d** Metabolite differences in females. Metabolites in black were decreased. Metabolites in red were increased. **e** Classifier performance by receiver operator characteristic (ROC) curve analysis. Z Z-score, FC fold change, *p* Student’s p value, q Bayesian q value.

Multivariate analysis showed that patients with treatment-refractory major depressive disorder were well separated from age- and sex-matched healthy controls by PLSDA metabolomic analysis (Fig. 2a). Pathway impact analysis showed that males and females with trMDD-SI had similar metabolic disturbances but prioritized different metabolic pathways (Fig. 2b). For example, males showed a greater reduction in plasmalogens related to peroxisomal metabolism. Females showed a greater increase in signaling eicosanoids like 5-hydroxeicosatetraenoic acid (5-HETE) (Fig. 2b). The top 30 discriminating individual metabolites are shown in Fig. 2c, d. Sphingolipids like ceramides and sphingomyelins, and several classes of phospholipids were decreased in both males and females. Seventy-six percent of the metabolic impact in females and 56% in males came from abnormalities in lipids (Supplementary Tables S1–S4). Seventy to 80% of the metabolic changes found were the result of decreased levels in trMDD-SI compared to controls (black labeled metabolites in Fig. 2c, d, Supplementary Tables S3-S6). Receiver operator characteristic (ROC) curve analysis showed that sets of just 5 metabolites could be used to classify patients with trMDD with over 90% accuracy and a sensitivity and specificity of over 85% (Fig. 2e). Pearson correlation analysis and multiple linear regression analysis showed that SIQ and BDI scores correlated with one another (r = +0.57, *p* < 0.0001, *q* < 0.002). However, strong metabolic predictors of self-reported affect, with *q* < 0.05, were not found (Supplementary Tables S7–S8).

### Mitochondrial abnormalities

**Fig. 3:**
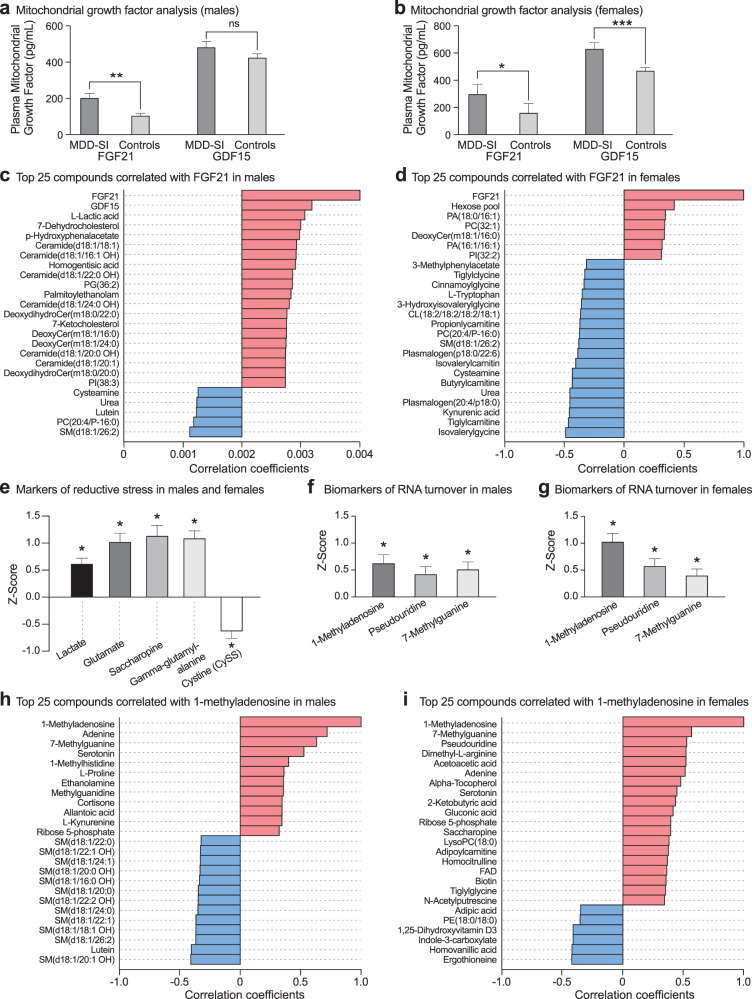
Biomarkers of mitochondrial dysfunction. **a** and **b** Plasma FGF21 and GDF15 in males (**a**), and females (**b**), **c** and **d** Metabolite correlations with FGF21 in males (**c**), and females (**d**)**. e** Biomarkers of reductive stress; increased NAD(P)H to NAD(P)+ ratio**. f** and **g** Biomarkers of RNA turnover in males (**f**), and females (**g**)**, h** and **i** Metabolite correlations with 1-methyladenosine in males (**h**), and females (**i**).

FGF21 and GDF15 levels were measured as biomarkers of mitochondrial dysfunction and the mitochondrial integrated stress reponse [47]. In males, FGF21 levels were increased in the trMDD-SI group. In females, both FGF21 and GDF15 were increased (Fig. 3a, b). When these mitokines were correlated with the rest of the metabolome, several sex-specific differences appeared. In males, FGF21 correlated with GDF15, lactate, and the cholesterol intermediate 7-dehydrocholesterol (Fig. 3c). In females, both FGF21 and GDF15 were increased and FGF21 was found to correlate positively with the plasma hexose pool (>95% glucose) levels and several PA and PC phospholipids (Fig. 3d). FGF21 is an established biomarker of reductive stress [48]. We found increased lactate, glutamate, saccharopine, gamma-glutamyl-alanine, and a corresponding decrease in the oxidized disulfide of cysteine, cystine (Fig. 3e), consistent with reductive stress. These metabolites result from a decrease in mitochondrial oxidation of NADH, which leads to an increase in the NADH/NAD+ and NADPH/NADP+ ratios in the cell. Mitochondrial 1-carbon and folate metabolism are dysregulated by the mitochondrial integrated stress response [49], and cerebral folate deficiency has been found in trMDD-SI [14]. In the current study, plasma 5’-methyltetrahydrofolic acid was decreased in females (Z-score = -0.76, *p* < 0.007) but not in males (Supplementary Table S6).

### Increased RNA turnover

Modified purines like 1-methyladenosine, and 7-methylguanine derived from tRNA, rRNA and mRNA turnover, and modified pyrimidines like pseudouridine from tRNA turnover were increased in both males and females (Fig. 3f, g). In males, 1-methyladenosine was negatively correlated with several sphingomyelin (SM) lipids and the carotenoid lutein (Fig. 3h). In females, 1-methyladenosine was negatively correlated with 1,25-dihydroxyvitamin D3 and ergothioneine (Fig. 3i).

### Metabolomics for personalized medicine

**Fig. 4:**
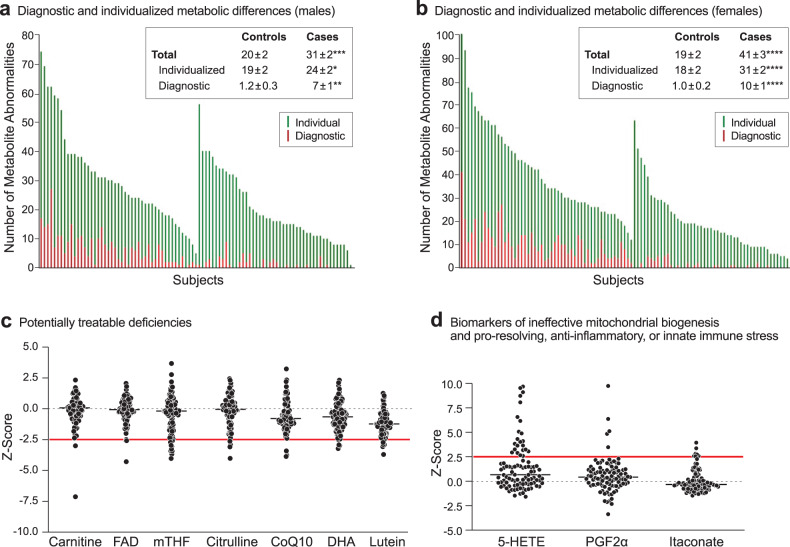
Metabolomic phenotyping provides information for both disease diagnosis and for personalized medical care. **a** and **b** Total and diagnostic metabolic abnormalities in patients with treatment-refractory major depressive disorder and healthy controls in males (**a**), and females (**b**). *p* values: *<0.05; **<0.01; ***<0.001; ****<0.0001. **c** 99% confidence interval analysis (≤ -2.5 Z-scores) identified individual patient deficiencies that are potentially treatable. **d** Biomarkers of ineffective mitochondrial biogenesis, anti-inflammatory, or innate immune stress (≥ +2.5 Z-scores).

Broad-spectrum metabolomic analysis permitted us to distinguish the subset of metabolites that was abnormal in trMDD-SI from the total number of metabolite abnormalities found in each patient. A metabolite abnormality was defined as a plasma concentration that was outside the 95% confidence interval (absolute Z-score > 2.0) found in health controls. We found that males with trMDD had an average of 31 ± 2 metabolites that were outside of the 95%CI, but only 7 ± 1 (22%) were found to distinguish cases from healthy controls (*p* < 0.05, VIP > 1.5). In females, there were an average of 41 ± 3 metabolites outside the 95%CI, with 10 ± 1 (24%) that were increased or decreased in trMDD compared to controls (Fig. 4a, b). This analysis showed that 75–80% of the abnormalities found were personalized differences, and 20–25% of the differences were diagnostic for group metabolic differences associated with trMDD-SI.

We next asked if there were any metabolic abnormalities that might permit personalized interventions. Using a Z-score of greater than +2.5 or less than -2.5 (99%CI) as the cutoff, we found individual patients with deficiencies in metabolites such as carnitine, FAD/riboflavin, methyl-tetrahydrofolic acid (mTHF), citrulline, CoQ10, docosahexaenoic acid (DHA), or lutein (Fig. 4c). Other patients with trMDD had increases in 5-hydroxyeicosatetraenoic acid (5-HETE), prostaglandin F2α (PGF2α), and itaconic acid (Fig. 4d), which have been associated with post-acute inflammation, pro-resolving, and innate immune effects [50].

## Discussion

The clinical spectrum of major depressive disorder is broad and only 30%-50% of patients respond well to first-line drug therapies directed at serotonin and norepinephrine neurotransmitters [51]. Metabolomic analysis provides a new tool for identifying novel mechanisms and biological targets that are likely contributors to the clinical heterogeneity of neuropsychiatric disease. Peripheral blood and cerebrospinal fluid (CSF) metabolomic studies are complementary. Recent studies have shown that CSF and plasma metabolomes are largely uncorrelated. Only about 0.6% of the possible correlations between blood and CSF metabolomes are statistically significant [52]. This is a natural consequence of the blood-brain barrier and underscores the biological fact that although influenced by the brain, peripheral blood metabolomic changes cannot yet be used to predict CSF abnormalities. Past studies have shown the promise of CSF metabolomics in identifying potentially treatable abnormalities such as cerebral folate deficiency in patients with treatment-refractory major depressive disorder [14]. The purpose of the current study was to investigate the potential of peripheral blood metabolomics for providing additional insights into the biology of depression with suicidal ideation, and to identify potentially treatable metabolic abnormalities.

### The mitochondrial nexus for stress signaling

Metabolomics revealed a convergence of many abnormalities in treatment-refractory major depressive disorder that were traceable to mitochondrial dysfunction. Mitochondria lie at the heart of the mitochondrial information processing system (MIPS) that regulates signaling between the brain and the periphery [53]. Mitochondria coordinate the cellular response to diverse environmental stresses [54]. This has been called the mitochondrial nexus [55]. Anterograde and retrograde communication between mitochondria and the nucleus is used to regulate gene expression via epigenetic changes [56]. A conserved transcriptional response to adversity (CTRA) has been documented in depression [57]. The genes regulated by the CTRA evolved originally as anti-microbial defense genes. Normal mitochondrial function is required to inactivate inflammatory oxylipins like 5-HETE and dampen the inflammatory response [58]. Recent work on the cell danger response has shown how programmed changes in mitochondrial function are used to complete three steps in the healing cycle after injury. When these steps are blocked by extracellular ATP release that is universally associated with stress-gated purinergic signaling, chronic illness results [59].

### Evidence for reductive stress with suicidal ideation

Changes in mitochondrial bioenergetic and redox functions have effects on nearly every class of lipid [60]. We found that 56%-76% of the metabolic impact in trMDD-SI involved lipid pathways. Well-known biomarkers of mitochondrial reductive stress like FGF21 and lactic acid were elevated. The overall pattern supported disturbances resulting from chronic mitochondrial reductive stress. Mitochondrial oxidative phosphorylation is regulated in part by purinergic signaling [61]. Extracellular ATP, ADP, and uridine nucleotides that are released through stress-gated cell membrane channels as a final common denominator in response to many kinds of environmental stress or infection [62]. After extracellular release, eATP is metabolized to ADP and adenosine, which act as ligands that activate ionotropic P2X and G-protein coupled P2Y and P1 adenosine receptors. In the brain, extracellular ATP release associated with endurance exercise acts to suppresses mitochondrial NADH oxidation, leads to coordinated increases in cerebrospinal fluid lactic acid, dopamine, and several other metabolites, and to a cascade of adaptive benefits that appear during recovery over the next several days [52].

While NADH and NADPH are abundant intracellular metabolites, they are not directly detectable in extracellular plasma using our methods. Despite this limitation, the chemical consequences of redox disturbances within the cell can be monitored by measuring the effects of reductive or oxidative stress in the plasma. Mitochondrial failure to oxidize NADH not only leads to an increased NAD(P)H/NAD(P)+ ratio in the cell and drives lactate production by lactate dehydrogenase (LDH) but also leads to an increase in several other molecules metabolized by mitochondria. For example, glutamate is normally converted to alpha-ketoglutarate and consumed by mitochondria in the Krebs cycle. When the Krebs cycle is inhibited by an increased NADH/NAD+ ratio, several Krebs cycle intermediates like alpha-ketoglutarate can build up in the cytoplasm. Increased saccharopine (epsilon-N-glutaryl-lysine) is a biomarker of increased NADPH and alpha-ketoglutarate. Saccharopine is synthesized by the NADPH-dependent enzyme, lysine oxoglutarate reductase [63]. Metabolomics revealed other evidence of reductive stress. For example, the oxidized disulfide of cysteine, L-cystine (CySS), was decreased in plasma compared to healthy controls. While more oxidizing conditions associated with a decrease in the plasma cysteine/cystine redox ratio are well-known risk factors for pro-inflammatory signaling and cardiovascular disease [64], studies of effects of reductive stress caused by decreased mitochondrial oxidation of NADH have recently highlighted how redox abnormalities in either direction can lead to disease [65].

The association of *reductive* stress as a metabolic feature of suicidal ideation in patients with refractory depression is a novel finding of this study. This contrasts with the *oxidative* stress associated with major depression in the absence of suicidal ideation [66]. Oxidative stress and reductive stress represent two poles of a spectrum that is controlled by mitochondrial function. Because all tissues are mosaics of cells that require different forms of mitochondrial function, it is possible to detect the signatures of both oxidative stress and reductive stress in the same blood sample. In the most severe forms of mitochondrial dysfunction, such as mitochondrial encephalomyopathy with lactic acidemia and stroke-like episodes (MELAS), *reductive* stress predominates [48]. When mitochondrial function decays, oxidative stress decays to reductive stress. This happens because active mitochondria are required to produce oxidants like superoxide and hydrogen peroxide. As mitochondrial membrane potential and oxygen consumption decline, mitochondria are unable to consume NADH fast enough to keep pace with its production, mitochondrial reserve capacity is exhausted, and the NADH/NAD+ ratio in the cell rises. The finding of reductive stress in suicidal ideation in this study underscores the importance of monitoring mitochondrial function as a predictor of suicide risk. FGF21 has emerged as a useful biomarker of mitochondrial dysfunction associated with reductive stress [48].

### Redox crosstalk between folate and biopterin metabolism

Up to 36% of patients with treatment-refractory major depressive disorder have been found to have cerebral folate deficiency, and one of the 33 patients studied had a combination of cerebral folate deficiency and cerebral tetrahydrobiopterin deficiency [14]. When folate or biopterin deficiency is found in the cerebrospinal fluid of patients with trMDD-SI, treatment with folinic acid or sapropterin (BH4), respectively, is often effective in improving symptoms when first-line therapies had failed [14]. Recent mouse studies of biopterin metabolism using knockouts of the gene for quinonoid dihydropteridine reductase (QDPR), have revealed unexpected connections between folate and biopterin metabolism [67]. QDPR encodes the NADH-dependent enzyme dihydropteridine reductase (DHPR), which is needed for redox regeneration of tetrahydrobiopterin (BH4) from dihydrobiopterin (BH2). Genetic knockout of QDPR produced a paradoxical decrease in tetrahydrofolic acid with normal levels of BH4. The authors suggested that the NADPH-dependent dihydrofolate reductase (DHFR) needed for folate recycling accepted BH2 as an alternative substrate when QPDR is mutated. The cited studies show that a number of different environmental stressors that lead to oxidative stress like mitochondrial toxic air pollutants [68] and most neuropsychiatric disorders studied [66], lead to mitochondrial dysfunction caused by the diversion of oxygen from oxphos to reactive oxygen species (ROS) production. If this continues chronically, mitochondrial reserve capacity is exhausted, and the cell enters a more severe state of mitochondrial dysfunction associated with reductive stress. These redox changes can lead in turn to secondary folate and/or biopterin deficiency, with associated neurological and psychiatric complications. When cerebral folate or cerebral biopterin deficiencies are found, treatment can result in significant clinical improvements [14, 69].

### Mitochondrial hypometabolism and RNA turnover

We found that 80% of the diagnostic metabolites in treatment-refractory major depressive disorders with suicidal ideation were decreased compared to healthy controls. In contrast, intermediates of RNA turnover like 7-methylguanine, 1-methyladenosine, and pseudouridine were increased. 1-methyladenosine (m1A) was most increased in trMDD-SI. This modification in mitochondrial mRNA is regulated by environmental stress and dynamically regulates mitochondrial protein translation [70]. Under conditions of mitochondrial dysfunction leading to hypometabolism and reduced metabolite flux, intracellular bioenergetics is impaired, and the redox- and energy-requiring steps needed for protein translation such as formyl-methionine synthesis, tRNA aminoacylation, and ribosome docking, and mRNA translation are slowed, and RNA turnover is increased. Several different causes of mitochondrial dysfunction lead to a mitochondrial integrated stress response (ISR) and secondary folate deficiency [49]. Recently, mitochondrial oxidative phosphorylation has been shown to be dependent on formyl-methionine synthesis that is used to initiate translation of mitochondrial respiratory chain mRNAs [71]. These results help explain the findings of folate deficiency and increased modified purines and pyrimidines derived from RNA turnover in the context of mitochondrial dysfunction in major depressive disorder.

### Purines and purinergic signaling

Abnormalities in purine metabolism and purinergic signaling have emerged as a common metabolic denominator in several neuropsychiatric disorders including MDD [72] and suicidal behavior [73]. Several purines like inosine, adenosine and guanosine, and pyrimidines like uridine were decreased in our cohort of patients with treatment-refractory major depressive disorder. A decrease in plasma purine and pyrimidine metabolites of purinergic signaling nucleotides like ATP and adenosine is consistent with hypersensitivity to extracellular purine signaling that induces the downregulation of ionotropic and G-coupled protein receptors (GPCR) used for purinergic signaling. A recent study found that the P2Y12 and P2Y13 receptor mRNAs were chronically decreased in patients with bipolar disorder and MDD either with or without non-violent suicide attempt but were increased back to neurotypical levels in patients after violent suicide attempt [74]. These results suggest the novel hypothesis that violent suicide attempts might be made in part to satisfy a physiologic impulse to decrease chronic cell danger signaling and restore normal purinergic signaling. Self-injurious behavior and problems with impulse control are well-established complications of excess purine production and signaling in the classical neurogenetic disorder Lesch-Nyhan syndrome [75].

The current study did not test purinergic signaling directly. However, mouse studies have shown that systemic injection of extracellular ATP (eATP), the canonical ligand for P2X receptors and the precursor to ADP and adenosine ligands of P2Y and P1 receptors, profoundly inhibited mitochondrial oxidative phosphorylation, decreasing energy production by as much as 74%, increased lactate, and led to coordinated changes in hundreds of other metabolites [61]. Antipurinergic drug therapy has been shown to restore normal levels of downregulated purinergic receptors like the P2Y1 GPCR in the Fragile X mouse model of autism spectrum disorder [76].

### Study Limitations

The generalizability of the main findings in plasma of stress-related mitochondrial dysfunction in severe depression are supported by a growing number of independent studies [77–79]. The metabolic profile of high suicidality in non-depressed patients, for example in PTSD without depression, or in other neuropsychiatric diagnoses associated with increased suicide risk was not examined in this study. The study did not include drug-naïve patients, treatment-responsive MDD patients, or patients with refractory MDD without suicidal ideation.

## Conclusions

Our results extend a growing number of studies that have shown the connection between mitochondrial dysfunction, purinergic signaling, depression, impulse control, and suicide [62, 74, 78, 80]. Metabolomic analysis of peripheral blood identified individual patients with deficiencies in carnitine, CoQ10, folic acid, citrulline, vitamin D, lutein, and other nutrients, metabolites and cofactors that might be targeted for personalized neuropsychiatric care. An increase in FGF21 was found in both males and females with suicidal ideation. Other biomarkers of mitochondrial dysfunction included abnormalities in sphingolipid and phospholipid metabolism, increased markers of mRNA and tRNA turnover, and the dysregulation of purine metabolism and purinergic signaling. These results identify new areas for basic research and add support for the growing interest in the development of anti-purinergic medications [81] and targeted metabolic interventions [14, 69] to improve the care of patients with major depressive disorder. Future studies will be needed to compare the relative clinical value of cerebrospinal fluid and peripheral blood metabolomics in the management of patients in whom first-line treatments have failed.

### Supplementary information

Supplementary Methods and Figure S1

Supplementary Tables S1-S9

## Data Availability

Raw AUC data from the LC-MS/MS analysis are provided in Supplementary Tables S1-S2.
