# Supplementary material for: Metabolic features of treatment-refractory major depressive disorder with suicidal ideation
Source: Transl Psychiatry. 2023 Dec 15;13:393. doi: 10.1038/s41398-023-02696-9 (PMC10721812; doi:10.1038/s41398-023-02696-9)

Supplementary Information

Metabolic features of treatment refractory major depressive disorder with suicidal ideation

Lisa A. Pan*[6,8,11,12], Jane C. Naviaux[1,5], Lin Wang[1,2], Kefeng Li[1,2], Jonathan M. Monk[1,2], Sai Sachin Lingampelly[1,2], Anna Maria Segreti[6], Kaitlyn Bloom[7], Jerry Vockley[7], Mark A. Tarnopolsky[10], David N. Finegold[8,11], David G. Peters[6,8,9,11], Robert K. Naviaux*[1-4]

The Mitochondrial and Metabolic Disease Center[1], Departments of Medicine[2], Pediatrics[3], Pathology[4], and Neurosciences[5], University of California, San Diego School of Medicine, 214 Dickinson St., Bldg CTF, Rm C107, San Diego, CA 92103-8467. University of Pittsburgh, School of Medicine, Department of Psychiatry[6], Department of Pediatrics[7], School of Public Health, Department of Human Genetics,[8] Department of Obstetrics, Gynecology & Reproductive Sciences[9], Pittsburgh, PA 15224. Department of Pediatrics, McMaster University, Hamilton, Ontario, Canada[10], Panomics Mental Health Initiative, Pittsburgh, PA 15228[11], New Hope Molecular, LLC, Pittsburgh, PA 15228[12].

SUPPLEMENTARY METHODS

Metabolomics

A total of 672 endogenous metabolites were targeted as previously described (1) with the following modifications. 563 metabolites of 672 (84%) were targeted by hydrophilic interaction liquid chromatography (HILIC) tandem mass spectrometry (LC-MS/MS) and 109 metabolites (16%) were targeted using reverse phase chromatography LC-MS/MS. Reverse phase (RP) tandem mass spectrometry was conducted using a Shimadzu LC-20AD UHPLC system coupled with a SCIEX Qtrap 5500 MS/MS as previously described for HILIC analysis (1). Ten µL of an 80% ethanol extract containing stable isotope-labeled internal standards was injected into the column through a CTC PAL autosampler. The samples were separated using a Raptor Biphenyl column (150 × 2.1 mm, 2.7 µm) (Restek). The LC conditions were as follows: Mobile phase A: 90% H_2O with 0.1% formic acid and 10% methanol (MeOH), pH 4.0. Mobile phase B: MeOH-Isopropyl alcohol (IPA) (50:50, v/v) with 0.1% formic acid. The gradient was: 0 - 2 min 10% B, 2.1 - 4 min 40% B, 4 -12 min, linear ramping up to 100% B, 12 – 18 min 100% B, 19 - 24 min 10% B, 24.1 min stop. The flow rate was 250 µL/min. The column temperature was controlled at

40ºC. The MS/MS detection was performed using electrospray ionization (ESI) and by advanced scheduled multiple reaction monitoring (MRM). The ESI source conditions were set as follows: electrospray voltage of -4500V for negative mode and 5500V for positive mode, source temperature of 500 °C, curtain gas of 30, ion source gas 1 and gas 2 of 35 psi, respectively.

Custom Synthesis of ^{13}C-labeled Standards in Pichia pastoris

Uniformly labeled stable isotope internal standards were custom-synthesized in the yeast *Pichia pastoris* (also called *Komagataella phaffii*, ATCC Cat# 76273) by metabolic labeling with $^{13}C_6$-glucose (Cat# 389374, Sigma Aldrich, 99.5% enrichment) and ^{13}C-sodium formate (Cat# 279412, Sigma Aldrich, 99% enrichment). After experimentation with several strains of *E. coli (e.g., NCM3722),* yeast (e.g., *S. cerevisiae BR-F diploid),* and worms (e.g., *C. elegans* N2), we found that the methylotrophic yeast *Komagataella phaffii*, formerly known as *Pichia pastoris*, could be grown to high yields in defined elemental medium. This permitted us to grown cultures in 1% (10 g/L) $^{13}C_6$-glucose and 1 mM ^{13}C-sodium formate in M9 minimal medium supplemented with trace element salts and biotin (10 ng/mL, 40 nM). Basic media recipes can be found in the book, *Pichia Protocols* (2), and the excellent paper by Neubauer et al. (3). We found that the addition of formate was needed to achieve ≥98% isotopic enrichment of purines and pyrimidines. Omission ^{13}C-formate resulted in cultures incorporating ^{12}C-CO_2 from the atmosphere for RNA and DNA synthesis, thereby decreasing the isotopic enrichment in purines and pyrimidines to below 90%, while carbon from $^{13}C_6$-glucose was used preferentially by the yeast to synthesize amino acids, lipids, and carbohydrates with enrichments >98%. Briefly, our protocol was to streak out a frozen glycerol stock of *Komagataella phaffii* (ATCC Cat# 76273) on a 1.5% Bacto agar (Difco cat# 214010) M9 minimal media plate supplemented with trace element salts (3) and biotin (10 ng/mL) and providing ^{13}C-glucose (1%) and ^{13}C-sodium formate (1 mM) as the only carbon sources. Isolated colonies were picked after growth at 30˚C for 3

days and used to seed 5 mL liquid minicultures. Minicultures were grown overnight at 30˚C at 200 rpm to an OD600 of 2-3. One miniculture was then used to seed a 100 ml maxiculture and grown overnight at 30˚C at 200 rpm to an OD600 of 3-4. Cells were harvested by centrifugation at 4000g at 4˚C and resuspended into a final volume of about 16 ml of M9 medium. One mL of the resuspended pellets contained about 0.2 g of cells and was distributed into each of 16, 1.7 ml microfuge tubes. These were pelleted at 10,000 rpm x 5 min and the supernatant above the cell pellets was removed. The 200 µL pellets were then frozen at -80˚C until extraction of ^{13}C-labeled metabolites. A 100 mL maxiculture typically produced 3.0-3.5 grams of cellular biomass. When ready for extraction, a tube with 200 mg of labeled and frozen *Pichia* cells was thawed, processed using 4 snap freeze-thaw cycles in liquid nitrogen and cold water then vortexed. Metabolites were then extracted by adding 4 volumes (800 µL) of cold (-20˚C) methanol-acetonitrile (50:50) to the 200 µL of freeze-fractured cells. The suspension was vortexed, incubated on ice for 10 min, vortexed again, then cellular debris and macromolecules were removed by centrifugation at 16,000g for 10 min at 4˚C. The supernatants containing ^{13}C-labeled metabolites were then transferred to brown glass vials and frozen at -80˚C until use.

Calibration of Custom-Synthesized ^{13}C-Labeled Standards

Each lot of custom-synthesized standards was calibrated by determining the percent ^{13}C enrichment of leucine, uridine, adenosine, adenosine monophosphate, glycerol-3-phophate, palmitoyl-carnitine, and selected phosphatidylcholine lipids and sphingomyelins. By using 5 µL of *Pichia* extract per 90 µL of plasma, one maxiculture of 3-3.5 grams of pelleted cellular wet weight typically provided enough ^{13}C-labeled standards for use as internal standards in the processing and extraction of over 2000 plasma samples.

Additional Chemical Standards

The Naviaux Lab Metabolomics Core at the University of California, San Diego (UCSD) maintains a library of 724 (and counting), purified standards for targeted broad-spectrum LC-MS/MS analysis. Compound-specific source and fragmentation parameters for 2-6 multiple reaction monitoring transitions (MRMs) for each targeted compound, along with MS/MS spectral data, and retention times by HILIC and RP separation were optimized using these purified standards. Commercial stable isotope standards for amino acids (Cat# NLM-1328 and CNLM-507), acyl-carnitines (Cat# NSK-B and NSK-B-G1), phospholipids (Cat# DLM-11099), and ceramides (Cat# CLM-9582) were purchased from Cambridge Isotope Lab (MA, USA) and used as internal standards.

Quality Control (QC)

Two levels of reproducibility were used daily before passing metabolomic results for data analysis—sample QC, and platform QC. In the first level, stable isotope-labeled internal standards were added to every biological sample, extracted, and the peak shape, retention time shift, and peak area variability of spiked stable isotope labeled internal standards were inspected in each sample. Samples that failed this QC analysis were reinjected the next day. If the same sample failed QC twice, it was excluded from further analysis. In the second level of quality control, HPLC and mass spec instrument stability were assessed using 4 replicate injections per day of a standardized lot of pooled human plasma containing stable isotope labeled internal standards (SIL-ISDs). The AUCs of 59 representative metabolites (13 SIL-ISDs and 46 endogenous compounds) in the replicate QC samples were monitored daily for platform process control. Reproducibility was quantified by calculating the within-day and within-day plus between-day Pearson correlations, and median of the relative standard deviations (RSDs). The intra-day and inter-day Pearson correlations for QC injections were typically ≥ 0.999 and 0.998, respectively. The intra-day and inter-day median RSDs for 59 representative metabolites in replicated QC injections on 3 days were 7.0% (IQR 4%-14%), and 6.0% (IQR 3%-13%),

respectively. If the Pearson correlation dropped to ≤ 0.98, or a daily median RSD increased to >15%, further injections were paused and system trouble shooting was commenced until the source of the platform instability was identified and corrected.

Statistical Analysis

Classifiers of 5-10 metabolites were selected and tested for diagnostic accuracy using area under the receiver operator characteristic (AUROC) curve and random forest analysis. Confidence intervals for the ROC curves were calculated by bootstrap resampling. Classifiers were validated within sample using repeated double cross validation (rdCV) (4), with bootstrapping 100 times to test random subsamples of 2/3 in and 1/3 out, and by permutation analysis (5). Results were organized into biochemical pathways and visualized in Cytoscape version 3.4.0. Metabolic network correlation analysis was performed by Pearson and Spearman analysis. Statistical methods were implemented in Stata (Stata/SE16, StataCorp, College Station, TX), Prism (Prism 9, GraphPad Software, La Jolla, CA), Python, or R.

SUPPLEMENTARY REFERENCES

1. Li K, Naviaux JC, Bright AT, Wang L, Naviaux RK (2017): A robust, single-injection method for targeted, broad-spectrum plasma metabolomics. *Metabolomics : Official journal of the Metabolomic Society*. 13:122.
2. Higgins DR, Cregg JM (1998): *Pichia protocols*. Totowa, New Jersey: Humana Press.
3. Neubauer S, Haberhauer-Troyer C, Klavins K, Russmayer H, Steiger MG, Gasser B, et al. (2012): U13C cell extract of Pichia pastoris--a powerful tool for evaluation of sample preparation in metabolomics. *J Sep Sci*. 35:3091-3105.
4. Filzmoser P, Liebmann B, Varmuza K (2009): Repeated double cross validation. *J Chemometrics*. 23:160-171.
5. Szymanska E, Saccenti E, Smilde AK, Westerhuis JA (2012): Double-check: validation of diagnostic statistics for PLS-DA models in metabolomics studies. *Metabolomics : Official journal of the Metabolomic Society*. 8:3-16.

Supplementary Tables

As a Single Excel File

Supplementary Table S1. Plasma metabolomics of males with treatment-refractory major depressive disorder and suicidal ideation. Raw data.
Supplementary Table S2. Plasma metabolomics of females with treatment-refractory major depressive disorder and suicidal ideation. Raw data.
Supplementary Table S3. Metabolic pathway analysis of treatment-refractory major depressive disorder and suicidal ideation. Ranked by random forest analysis. Males.
Supplementary Table S4. Metabolic pathway analysis of treatment-refractory major depressive disorder and suicidal ideation. Ranked by random forest analysis. Females
Supplementary Table S5. Plasma metabolomic analysis of males with treatment-refractory major depressive disorder and suicidal ideation.
Supplementary Table S6. Plasma metabolomic analysis of females with treatment-refractory major depressive disorder and suicidal ideation.
Supplementary Table S7. Metabolome correlations with SIQ and BDI scores. Males. Top 12 predictors.
Supplementary Table S8. Metabolome correlations with SIQ and BDI scores. Females. Top 12 predictors.
Supplementary Table S9. Medications in patients with treatment-refractory major depressive disorder and suicidal ideation.

Supplementary Figure

Supplementary Figure S1. Cytoscape maps of metabolic pathway impacts in treatment-refractory major depressive disorder with suicidal ideation. **A.** Males, **B.** Females. Percentages reflect the pathway impact calculated by random forest analysis. N = 47 trMDD-SI males and 46 male controls. N = 52 trMDD-SI females and 49 controls.

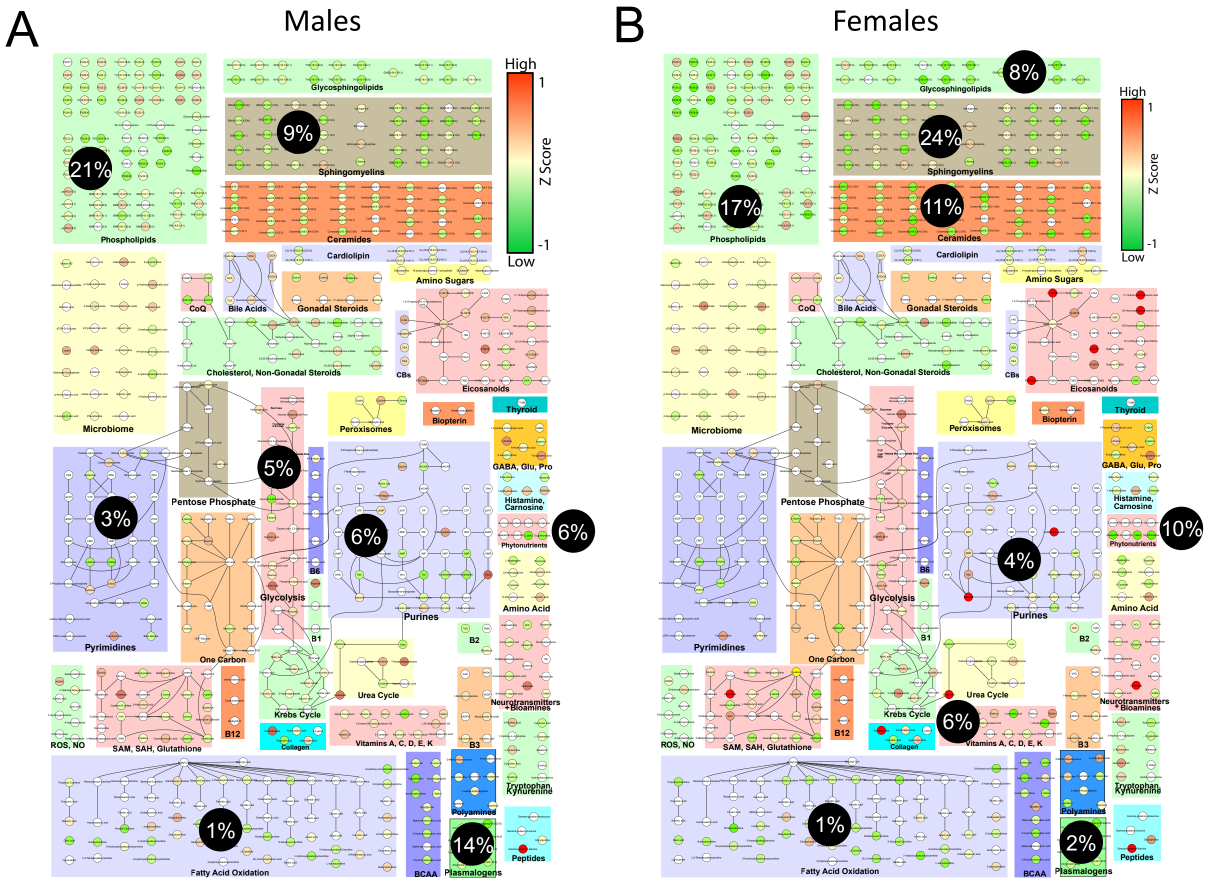

Supplement: Supplementary file 1 — Supplementary Methods and Figure S1 [file 41398_2023_2696_MOESM1_ESM.pdf]
